# Lead Catalyzed GaAs Nanowires Grown by Molecular Beam Epitaxy

**DOI:** 10.3390/nano14231860

**Published:** 2024-11-21

**Authors:** Igor V. Shtrom, Nickolai V. Sibirev, Ilya P. Soshnikov, Igor V. Ilkiv, Evgenii V. Ubyivovk, Rodion R. Reznik, George E. Cirlin

**Affiliations:** 1 Faculty of Physics, St. Petersburg State University, Universitetskaya Emb. 13B, 199034 St. Petersburg, Russia; 2 Department of Nanotechnology Methods and Instruments, Institute for Analytical Instrumentation of Russian Academy of Sciences, 198095 St. Petersburg, Russia; 3 Department of Epitaxial Nanotechnologies, Alferov University, 194021 St. Petersburg, Russia; 4 Division of Physics of Dielectric and Semiconductors, Ioffe Institute, 194021 St. Petersburg, Russia; 5 Institute of Advanced Data Transfer Systems, ITMO University, 197101 St. Petersburg, Russia

**Keywords:** semiconductor nanowires, vapor–liquid–solid growth, catalyst, doping, growth modeling, structural characterization, crystal phase

## Abstract

This study investigates the growth of gallium arsenide nanowires, using lead as a catalyst. Typically, nanowires are grown through the vapor–solid–liquid mechanism, where a key factor is the reduction in the nucleation barrier beneath the catalyst droplet. Arsenic exhibits limited solubility in conventional catalysts; however, this research explores an alternative scenario in which lead serves as a solvent for arsenic, while gallium and lead are immiscible liquids. Liquid lead easily dissolves in Si as well as in GaAs. The preservation of the catalyst during the growth process is also addressed. GaAs nanowires have been grown by molecular beam epitaxy on silicon Si (111) substrates at varying temperatures. Observations indicate the spontaneous doping of the GaAs nanowires with both lead and silicon. These findings contribute to a deeper understanding of the VLS mechanism involved in nanowire growth. They are also an important step in the study of GaAs nanowire-doping processes.

## 1. Introduction

AIIIBV nanowires (NWs) are a promising material for use in opto- and nanoelectronics devices [1,2]. Also, recently, AIIIBV NWs have demonstrated their importance in modern nanophotonics [3,4]. For example, single-photon sources could be constructed from GaAs quantum dots embedded in (Al, Ga)As NWs, as well as from crystal-phase quantum dots in NWs [5,6,7]. Heterostructured (Al, Ga)As NWs have great promise as a building block for modern cryptography and photonic quantum information technologies [4,5]. The capability of electric pumping is crucial to have, in order to ensure the efficient use of such structures. Thus, the practical application of GaAs NWs requires precisely controlled doping, both p- and n-type.

In general, heterostructured (Al, Ga)As NWs are formed via the so-called vapor–liquid–solid (VLS) mechanism [8,9,10]. The presence of a liquid droplet introduces complications to the growth and doping of heterostructures that are not present in bulk growth. One significant challenge is the inability to use Si or Sn for n-type doping [11,12,13]. Typically, Si and Sn produce p-type doping in GaAs in VLS growth [11].

The second problem is the so-called reservoir effect [10,14,15] attributed to the solubility of one or more growth species in the droplet. The reservoir effect has the effect of blurring heterointerfaces [14,15].

The first challenge could be solved using a group VI element for n-type doping [11]. These elements are volatile and violate the conditions of the ultra-high vacuum. Here, an alternative solution has been proposed.

The growth of NWs usually occurs in a nonequilibrium mode [8,9]. The composition of the NW and the incorporation of impurities is determined by kinetics rather than thermodynamics. The impurity embedded in the lattice kinks turns out to be covered by the following layers of material, and does not have time to return to the solution. Therefore, the place of the incorporation of the impurity is determined by the presence of free lattice kinks in the layer. In this case, the composition of the NWs and the incorporation of amphoteric impurities into the NWs is determined by the composition of the catalyst droplet. Most often, a drop of catalyst contains a lot of gallium and little arsenic [12]. If there is a lot of Ga in the droplet, then it is easier for silicon to incorporate into GaAs in place of As. That is, Si will be the acceptor. It is natural to expect that if there is a lot of As in the drop, then Si will be embedded in the place of Ga and will be a donor.

There are not many materials that dissolve As well and are liquid at typical GaAs NW growth temperatures. Some of them are poisonous, such as thallium. Interesting examples of such materials are tin (Sn) and lead (Pb), which could easily dissolve each of the elements Ga and As, but not simultaneously [16,17,18,19].

The growth of GaAs NWs with a Sn catalyst in both modes has already been demonstrated. Arsenic-rich growth has been demonstrated in Lund University [20]. Gallium-rich growth has been demonstrated in Ioffe institute, St. Petersburg [21]. In all cases of GaAs growth, NW with a Sn catalyst led to a higher level of contamination of NW by the catalyst material [22]. The binary and triple-phase diagrams of Pb with Ga-As and Sn with Ga-As are very similar. It can be expected that the growth of GaAs NWs with a Pb catalyst is also possible. The incorporation coefficient for Pb in GaAs is less than for Sn in GaAs. Therefore, it can be expected that the growth of GaAs NWs with Pb will be more stable, and the Pb contamination in GaAs NWs will be less than Sn. The reservoir effect problem can also be partially solved by growing in an As-rich regime. When growth (Al, Ga) As NWs occurs in an As-rich mode, the concentration of both group III elements in a droplet is very low. The GaAs/AlAs group III heterojunction becomes sharp without additional effort.

## 2. Materials and Methods

**Figure 1:**
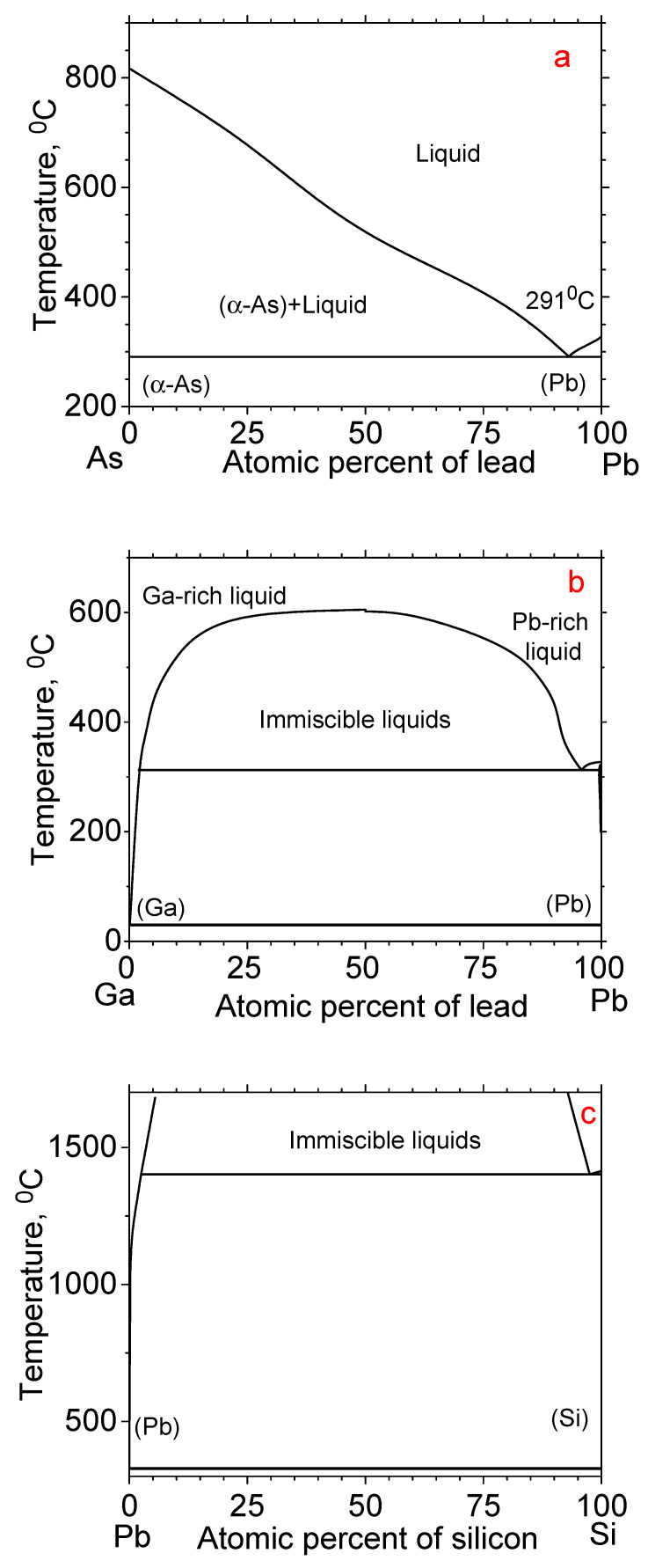
As-Pb (**a**), Ga-Pb (**b**), and Pb-Si (**c**) phase diagrams generated using the data from papers [16,19,23].

This study examines the growth of gallium arsenide (GaAs) nanowires using lead as a catalyst. The growth conditions were chosen for synthesis on an arsenic-rich catalyst. So, we chose conditions under which the solubility of arsenic in a lead liquid exceeds 20%, while the solubility of gallium is less than 10%; see Figure 1. The non-miscibility gap of lead and gallium disappears at a temperature of 600 °C [16]. Lead dissolves less than 10% of gallium at a temperature of about 400 °C and below [16]. Therefore, the growth temperature should be below or around 400 °C. The eutectic temperature of arsenic and lead is 291 °C [19], while the liquid contains only 7% arsenic. The solubility of arsenic in liquid lead reaches 20% at a temperature of about 350 °C and above [19]. Therefore, the growth temperature should be above 350 °C. Therefore, all growth processes took place in the temperature range from 350 °C to 410 °C.

GaAs nanowires (NWs) were grown on Si (111) with a predeposited thin lead layer in the solid-source molecular beam epitaxy (MBE) system Riber Compact 21 (Paris, France), equipped with effusive sources of gallium and arsenic.

First, the substrate was treated with a weak hydrochloric acid solution to remove the defective oxide layer. Then, a 10 nm thick lead film was applied using a BOC Edwards Auto 500 (Burgess Hill, UK) thermal resistance evaporator with oil-free injection and residual vacuum of at least 5 × 10^−6^ Torr, at a substrate temperature of about 80 °C. The thickness of the film was controlled by the transmission of light on the satellite glass. The optical circuit included a monochromatic light source (in our case, a 650 nm laser), a beam splitter, and control and measuring photodetectors. The source and receivers operated in pulse mode, which eliminates the influence of backlight. The thickness was determined according to the Lambert–Bouguer–Lambert extinction law (the exponential dependence of the signal on thickness). The thickness of the half-transmission was determined previously on a plate with a known thickness of the order of 100–200 nm.

Next, the substrate was transferred to the growth chamber of MBE system. The substrate was heated to a growing temperature in the range from 350 °C to 410 °C. Even a short-time annealing of the substrate leads to the dissolution of lead in silicon, which prevents the formation of lead catalyst droplets. NW growth without initial droplets at such temperatures doesn’t proceed. Therefore, the annealing of substrate wasn’t done, and oxide layer wasn’t desorbed. 

The shutters were opened simultaneously, and NW growth began immediately after growth temperature was reached. The gallium deposition rate was 0.7 ML/s. The total growth time was 10 min. The arsenic flux was ten times higher than the gallium flux. The flux calibration was based on the reflection high-energy electron diffraction (RHEED) on the surface of GaAs (100). The growth was stopped by simultaneously shutting down the fluxes and cooling the substrate.

NW shape studies were performed with a field-emission scanning electron microscope (SEM) Supra 25 (C. Zeiss, Oberkochen, Germany) operated at 20 kV, equipped with the microanalysis tool Ultim (Oxford Instruments Inc., Abingdon, UK) for energy dispersive spectrometry (EDS). An investigation of the structural properties and the composition of NWs were conducted by methods of a transmission electron microscopy (TEM) on Zeiss Libra 200FE microscope (Oberkochen, Germany), equipped with an energy-dispersive X-Max X-ray detector (EDX). The samples for TEM were obtained by depositing NWs onto carbon-film-coated copper grids by gently rubbing the grid against the sample, in most cases breaking the NWs off at the base.

## 3. Results and Discussion

**Figure 2:**
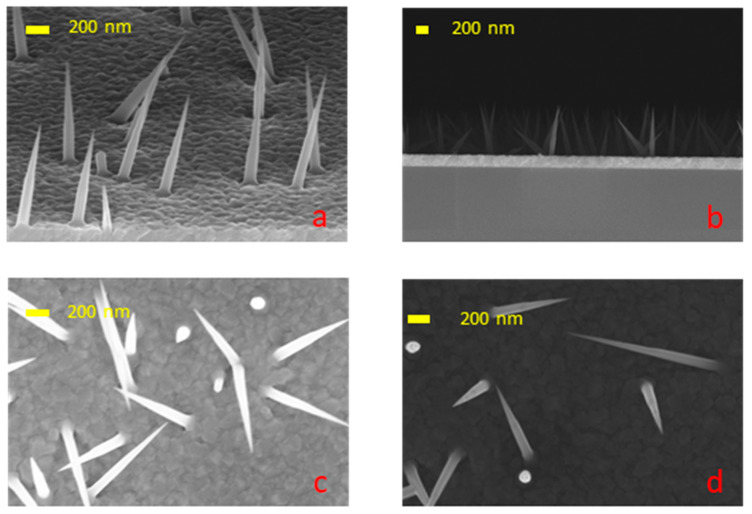
SEM images of NWs grown at different temperatures (**a**,**b**) 350 °C; (**c**) 380 °C; (**d**) 410 °C.

The typical array of NWs grown under the aforementioned conditions are shown in Figure 2. The obtained results demonstrate the growth of freestanding NWs. The NWs exhibited a conical shape, with the exception of the low-temperature sample. This shape is attributed to the incorporation of Pb in GaAs. At a low temperature, some NWs displayed a flat facet or particle on the tip. At all temperatures, the NWs are misoriented, most likely due to the oxide layer. As shown previously, annealing is necessary to obtain well-oriented nanowire arrays [24,25,26]. In our case, this was not possible, since long-time annealing causes the dissolution of the lead in the wafer.

The EDS measurements confirmed that the majority of the NWs are GaAs, with the potential presence of Si. Traces of Pb as well as Si were found in all NWs by using the EDX detector of TEM.

**Figure 3:**
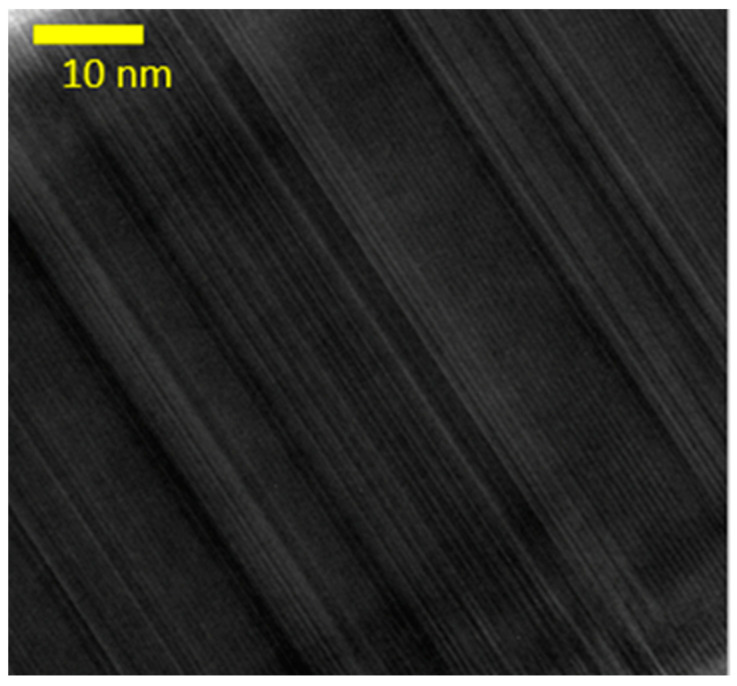
TEM image of nanowire grown at 350 °C.

The typical results of TEM studies are presented in Figure 3, which reveals that the NWs exhibit a polytypic structure, consisting of both the wurtzite and sphalerite phases. This observation applies to all analyzed samples and has also been confirmed by RHEED. It is likely that the unusually low growth temperatures (350–410 °C) for GaAs NWs are responsible for a polytypic structure.

Elemental analysis by EDX on samples grown at high temperatures (380 °C, 410 °C) revealed a significant presence of silicon, evenly distributed throughout the NWs. Single-point EDX analysis shows traces of silicon, while signals collected from large areas give values in range 0.05–2%. This allows us to conclude that silicon concentrations reached levels between 10^19^ and 10^2^ cm^−3^. However, lead in NWs grown at high temperatures were not detected, even when col-lecting the EDX signal from the NW stack on the TEM grid, with a scan area of 200 × 200 nm. This means that the doping level achieved with lead is below 10^18^ cm^−3^. It is most probable that Pb diffuses to the substrate surface and dissolves in silicon.

**Figure 4:**
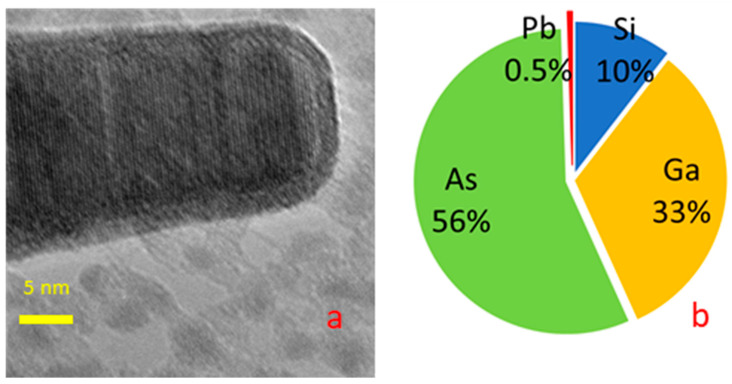
TEM image of nanowire tip grown at 350 °C (**a**) and its composition according to EDX measurements (**b**).

An EDX analysis of samples grown at a lower temperature (350 °C) yielded more interesting results. Lead was unambiguously detected by single-point EDX analysis in both cases in the body and tip of the NWs. The TEM image of the NW tip showed clear elemental contrast; see Figure 4a.

**Figure 5:**
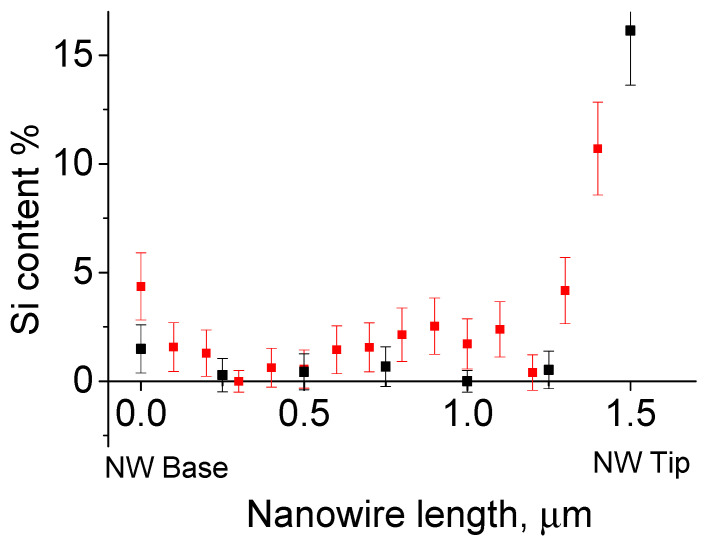
Si content along the NW length for different nanowires grown at 350 °C, first NW—black, second NW—red.

The silicon concentration within the NWs showed a remarkable variation. The silicon concentration ranged from about 5% at the base to almost the limit of sensitivity in the middle, and then reached high values of more than 10% at the NW tip; see Figure 5. It can be reasonably concluded that the silicon concentration within the body of the NWs is in the order of a few percent. Silicon concentrations measured at the NW tip ranged significantly from 5% to 20% depending on the tip morphology. The lead content remained minimal in all cases, see Figure 4b.

The arsenic clearly predominates over gallium at the tip of the NW; see Figure 4b. Moreover, the arsenic content in the upper part exceeds 50%. There is practically no lead detected at the top, and the solubility of arsenic in lead is limited. The lead content detected at the top of the nanowire is near the sensitivity threshold of the energy-dispersive X-Max X-ray detector. In some cases, lead lines even fall below the threshold. Pure arsenic desorbs from GaAs at the growth temperature used in our study. A possible explanation of high arsenic content, see Figure 4b, is the presence silicon arsenide’s SiAs and SiAs₂ at the nanowire tip. That is, the NW tip likely consists of silicon arsenide. This makes it possible to suppose that the NW catalyst was not a liquid solution of As and Si in a lead–gallium droplet, but a solid silicon arsenide particle with Ga and Pb impurities.

In the case of MBE NW growth, the catalyst at the tip primarily reduces the surface energy and lowers the nucleation barrier. Perhaps the lead at the NW tip acts as a chemical catalyst. Lead accelerates one of the following chemical reactions: 2SiAs + As₂ → 2SiAs₂ or SiAs₂ + Ga → SiAs + GaAs. It is noteworthy that such catalyst behavior has not been described, even in cases of GaAs NW growth with an arsenic-rich catalyst [20,27]. This hypothesis requires further verification. Future investigations of GaAs nanowire growth with Pb catalyst are planned at temperatures near or even below the eutectic point of lead and arsenic to better understand the underlying mechanisms.

**Figure 6:**
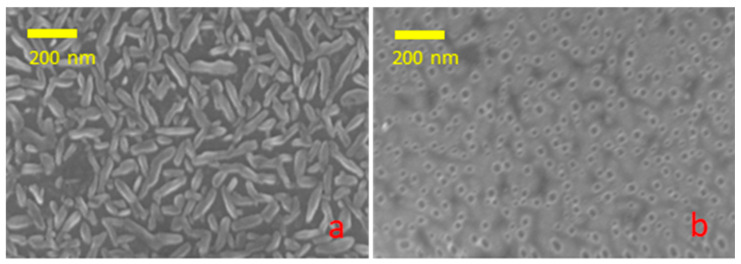
SEM image of Si substrate after thick lead film deposition (**a**) and after annealing (**b**).

Liquid lead easily dissolves in Si as well in GaAs. It is well known that lead and silicon form a continuous series of solid solutions. Thus, the annealing of lead thin-film on silicon results in the dissolution of lead in the substrate rather than droplet formation. The films, with a thickness of 1–3 nm, underwent dissolution in silicon when heated to 350 °C and subsequently cooled. A 30 s annealing period under an arsenic flux of a 10 nm film at a temperature of 300 °C yielded comparable results. The sole remaining evidence of the lead is the etching holes, as illustrated in Figure 6. NW growth is not initiated on these substrates. While the solubility of lead in GaAs is less than that in silicon, the issue of preserving the catalyst remains a significant concern. The consumption of lead during the growth process results in the formation of cone-shaped NWs and uncontrollable doping.

So, long-period annealing is not possible in our case of growth on Si substrate, and the growth of well-oriented Pb-catalyzed GaAs remains a challenge. Since well-oriented arrays require the removal of oxide [24,25,26], the Pb dissolves in GaAs slower. We expect that growth on GaAs substrate will allow us to remove the oxide layer from the GaAs substrate with Pb film and grow well-oriented NWs.

Our studies indicate the spontaneous doping of the GaAs nanowires with both lead and silicon. The silicon content in the body of GaAs NWs at low temperatures (350 °C) reaches 5%. The silicon is easily visible in single-point EDX as well as in linescan EDX, and even in EDS measurements. As the temperature increased, the silicon content decreased. A detectable Si signal below 0.5% from NW grown at 410 °C was collected only from the NW stacks on TEM grid. The active lead-etching of silicon substrate was observed; see Figure 6. Also, silicon incorporation into GaAs NWs was observed; see Figure 5. This effect disappears with the increase in temperature. Yet, the As-rich growth of NW is impossible at higher temperatures. The Ga-Pb miscibility gap becomes narrower with increasing temperature and closes near 600 °C. At the moment, the study of growing GaAs NW with a Pb catalyst on a GaAs substrate is in progress.

## 4. Conclusions

To conclude, the cumulative data presented in this paper support the following three related points. First, lead can readily act as a catalyst for GaAs NW growth, at least in the temperature range of 350–410 °C. Second, lead slowly dissolves in the GaAs NWs during the growth. The droplets gradually diminish in size. This process results in the formation of cone-shaped nanowires and, finally, could stop NW growth. Third, lead reduces the incorporation barrier of Si in GaAs nanowires. This could subsequently result in the formation of a Si-GaAs alloy. The potential role of lead as a catalyst within an arsenic-rich environment presents a novel perspective that merits further exploration. These findings contribute to a deeper understanding of the VLS mechanism involved in nanowire growth. We hope that further studies of the GaAs NWs growth in the arsenic-rich droplet mode will make it possible to select growth parameters for controlled doping with n-type impurities, which will expand the functionality of using GaAs NWs in various applications.

## Data Availability

Data are contained within the article.
